# Exercise testing and training in frail older adults with an orthopedic impairment participating in a geriatric rehabilitation program: an international Delphi study

**DOI:** 10.1007/s41999-023-00819-5

**Published:** 2023-07-04

**Authors:** Wim G. Groen, Elizabeth M. Wattel, Aafke J. de Groot, Franka J. M. Meiland, Cees M. P. M. Hertogh, Karin H. L. Gerrits

**Affiliations:** 1 Department of Medicine for Older People, Amsterdam UMC, Vrije Universiteit Amsterdam, De Boelelaan 1117, 1081 HV Amsterdam, The Netherlands; 2 Aging & Later Life, Amsterdam Public Health Research Institute, Amsterdam, The Netherlands; 3 Amsterdam Movement Sciences, Ageing & Vitality, Rehabilitation & Development, Amsterdam, The Netherlands; 4 Department of Human Movement Sciences, VU University Amsterdam, Amsterdam, The Netherlands; 5 Merem Medische Revalidatie, Hilversum, The Netherlands

**Keywords:** Geriatric rehabilitation, Exercise, Fractures, Delphi study

## Abstract

**Background:**

Little is known about exercise testing and training in orthopedic geriatric rehabilitation. This study aims to obtain expert-consensus-based recommendations on this matter.

**Methods:**

Using an online Delphi study we aimed to reach international expert consensus on statements related to testing and training of endurance capacity and muscle strength. Participants needed to have relevant research or clinical expertise. Statements were evaluated and explanatory comments could be provided. After each round anonymous results were presented to participants. Statements could be adjusted or new ones could be formulated if necessary. Consensus was defined as > 75% of participants agreeing.

**Results:**

Thirty experts completed the first round. Twenty-eight (93%) the second and 25 (83%) completed the third round. The majority of experts were physical therapists. Consensus was reached on a total of 34 statements. The statements and comments reflected the need for a pragmatic and tailored approach in this population both for testing and training. For example, for testing endurance capacity, a 6 Minute walk test was promoted and for testing muscle strength, performance in a functional activity was suggested. Ratings of perceived exertion were promoted for monitoring intensity of endurance and muscle strength training in patients without cognitive impairment.

**Conclusion:**

In orthopedic GR, endurance and muscle strength testing should be pragmatic and is preferably performed in functional activities. For endurance training existing guidelines of the American College of Sports Medicine can be strived for but adapted as needed and for muscle strength training only lower intensities are agreed upon.

**Supplementary Information:**

The online version contains supplementary material available at 10.1007/s41999-023-00819-5.

## Key summary points

**Aim:**

To obtain expert-consensus-based recommendations for exercise testing and prescription for orthopedic geriatric rehabilitation.

**Findings:**

For endurance training existing guidelines of the American College of Sports Medicine can be strived for but adapted as needed and for muscle strength training only lower intensities are agreed upon.

**Message:**

In orthopedic geriatric rehabilitation, endurance and muscle strength testing should be pragmatic and is preferably performed in functional activities.

**Supplementary Information:**

The online version contains supplementary material available at 10.1007/s41999-023-00819-5.

## Introduction

Geriatric Rehabilitation (GR) is defined as a multidimensional approach of “diagnostic and therapeutic interventions, with the purpose to optimize functional capacity, promote activity and preserve functional reserve and social participation in older people with disabling impairments” [1]. The largest subgroup of patients (about 40%) in GR are those who are admitted for an orthopedic problem as a result of a trauma (acute orthopedic) or elective surgery related to the lower extremities and pelvis [1]. We will refer to this group of group as orthopedic GR patients. To improve functional performance, these patients often engage in exercise training supervised by physical therapists as part of multidisciplinary treatment [2]. The focus of this exercise training is on regaining functional independence through practicing functional activities so that a patient is able to return home as soon as possible. Because of this focus on physical functioning, strength and endurance training are not always at the forefront of the GR trajectory. Strength and endurance training are part of the total rehabilitation trajectory, but the content varies in practice and is often not based on evidence based guidelines, which may lead to suboptimal functional outcomes. This may be partly caused by the lack of guidelines for specific patient populations. In addition, also generic guidelines are not always adhered to. In the field of GR, therapists seem not fully aware of the potential of strength and endurance training for improving activities of daily living. However physical functioning is highly dependent on physical fitness [3, 4] making it an important target.

There have been some recommendations for exercise training in older adults [5–7] as well as results from meta-regression analyses resulting in “optimal” training characteristics for endurance [8] and muscle strength training [9] in older healthy adults. One could question why these recommendations could not just be followed for orthopedic GR patients. First, the orthopedic GR population concerns a specific group of patients that is very heterogeneous with many comorbidities, frailty and disability [10]. Second, although a number of studies have been performed in frail older adults post fracture, their exercise programs are highly heterogeneous regarding their exercise programming and descriptions of exercise programs are often poorly reported [11–13]. Consequently, there is also a lack of evidence on how to set (based on valid exercise testing) and monitor training intensities for frail older adults in general and thus also for orthopedic GR patients.

The absence of evidence based guidelines inevitably leads to variation in daily clinical practice with regard to Frequency, Intensity, Type and Time (FITT) characteristics used in training and therefore to suboptimal treatment (i.e. under- or overtreatment). The art of exercise training is that it should be performed at the proper intensity and duration. This induces physiological overload and triggers adaptation as a result of super compensation during the subsequent resting period. Undertreatment (i.e. training at lower than optimal intensity or volume) may lead to suboptimal gains in physical fitness and -functioning whereas overtreatment (training at higher than optimal intensity or volume) may lead to further aggravation of the impairment or to adverse events. To be able to evaluate the exercise program and to train at the right intensities, proper evaluations should be made of (baseline) physical fitness by valid and reliable exercise testing methods.

To date there is a lack of evidence regarding exercise testing and training requirements (e.g. which tests to use, and how and which training characteristics to apply) in orthopedic GR patients. Therefore we performed a Delphi study to obtain expert- consensus based practical guidelines for exercise testing and prescription for these patients.

## Methods

### Study design

An online Delphi procedure was performed to reach consensus on exercise programming characteristics during orthopedic geriatric rehabilitation in frail older adults. Consensus or “collective agreement” is widely regarded as a useful method in the absence of scientific evidence. The objectives of the study were presented to all participants and informed consent was obtained before the start of the first Delphi round. The study did not need institutional review board approval as by Dutch law this type of research is exempted from review. All invited participants also provided consent to be acknowledged in this article.

### Expert panel

International experts were selected by three Delphi moderators (W.G., L.W. and K.G.). Experts needed to either have extensive knowledge of exercise physiology in frail older patients or extensive practical experience in exercise testing and training in frail older patients with an orthopedic impairment. Specifically, the experts needed to fulfill at least one of the following criteria 1) having a track record of at least 5 research publications in the field of exercise testing or training in frail older persons (preferably orthopedic rehabilitation), 2) having at least 5 years of recent experience in the field of exercise testing and training in orthopedic geriatric rehabilitation either as physiotherapist or as a physician, 3) having expertise, otherwise verifiable regarding the topic of exercise testing and training in orthopedic GR (e.g. educational experts on the topic). Participants were not informed about the identity of other participants in the study until completion of the study. Academics were primarily contacted via email as identified by publications or via a google search and Dutch geriatric PTs via the Dutch collaborative academic networks of elderly care (SANO). Additionally, some experts were approached via Twitter as a first contact. In all instances, when a first contact was established interested participants were sent a formal invitation letter by email with the purpose and procedures of the study.

### Delphi process

#### General process

The Delphi process comprised three rounds. We used Survalyzer software (Survalyzer BV, Utrecht, The Netherlands) to create the online questionnaires and to present feedback to the participants. All materials throughout the entire study were made available in both English and Dutch. The first questionnaire was sent in May 2021 and the deadline for filling out the third questionnaire round was August 2021. In case a panel member did not respond a reminder email was sent. We sought to reach consensus on the following four selected topics: (1) testing endurance capacity, (2) training endurance capacity, (3) testing muscle strength, (4) training muscle strength. Statements were developed by the moderators (W.G., L.W. and K.G.), based on their expertise and literature review on this topic. For example, with regard to FITT characteristics for muscle strength training we used the recommendations from Borde et al. [9]. A background information document and the questionnaire that were used in round one (Online Resources 1 and 2 respectively), were tested and revised for content, clarity, and lay-out by a clinical exercise scientist, a geriatric physical therapist and an elderly-care physician involved in geriatric rehabilitation.

In each round participants were asked to independently evaluate the statements using a 4-point Likert scale (’strongly agree’, ‘agree’, ‘disagree’, ‘strongly disagree’), or the option to select ‘don’t know’. A free-text response option was available for every statement to elaborate on or explain the responses. Lastly, participants were given the opportunity to provide a general comment per topic and for the questionnaire as a whole. Consensus was defined as > 75% of participants scored “agree” or “totally agree” on a statement in any of the rounds [14] (including potential “I don’t know” responses in the denominator). All statements on which consensus was reached were omitted in the second round. Statements with insufficient consensus were rephrased by the research team making use of the comments that were provided by the Delphi panel members. The Delphi study was finished after a predefined number of three rounds even if there were remaining statements for which consensus was not yet reached.

#### Round 1

The main part consisted of the above mentioned 4 topics. In total 29 statements were presented. Additionally, 8 closed and 10 open-ended questions were posed to gain better understanding on several topics (e.g. about the feasibility and validity of several methods for monitoring the intensity of endurance training) and to use for further construction of statements. In this round we also made an inventory of the sociodemographics and professional experience of the participants to describe the sample. The questions and statements posed in the three rounds are presented in Online Resource 2.

#### Rounds 2 and 3

Apart from rephrasing the statements where consensus was not reached, the moderators created new statements based on the input that was provided in round 1 on the closed and open ended questions and these were presented for the first time in round 2. We showed the panel members the results of the statements in the first round in a bar chart which were accompanied by the anonymous comments that were provided in the free text response option by the Delphi members. Responses were translated by the research team to both Dutch and English. When the moderators team felt that the feedback was very lengthy for a particular statement then a short summary was added as an introduction to the new statement. The procedures for round 3 were similar to those described for round 2.

### Data analysis

Descriptive statistics were used to describe participants’ demographic characteristics and responses to each statement in all three rounds.

## Results

In total 30 experts participated in the first round. Fourteen researchers were invited by email of which 11 agreed to participate and 8 completed the first round (participation rate of 57%). Three actively declined (2 because of limited time and one because of illness) and 3 that initially agreed in the end did not participate. The call for participation of physical therapists yielded 19 participants (participation rate unknown). Furthermore two elderly care physicians working in geriatric rehabilitation and one lecturer in geriatric exercise physiology were invited and all participated (100% participation rate). Twenty-eight (93%) completed the second and 25 (83%) completed the third round. The proportion of academics versus clinicians was fairly constant across the rounds. In the first round the fraction of researchers was 8/30 (27%), and was 7/28 (25%) and 5/25 (20%) in the second and third round respectively. The characteristics of the Delphi panelists are presented in Table 1.

**Table 1:** Characteristics of Delphi panel experts

Main profession, (*n =*)	
Physical therapist	19
Researcher	8
Elderly care physician	2
Other, teacher in geriatric exercise physiology	1
Country of residence, (*n =*)	
Australia	1
Canada	2
Denmark	1
Netherlands	17
Spain	1
United Kingdom	5
USA	3
Years of experience (scientific or clinical) regarding physical testing and or training elderly patients in general, mean (sd); range	18 (10); 5–38
Years of experience (scientific or clinical) regarding physical testing and or training elderly patients who are rehabilitating from an orthopedic procedure, mean (sd); range	15 (11); 0*–38

*Teacher in geriatric exercise physiology

### Evolution of statements throughout the study

In round 1, a total number of 29 statements were presented. Consensus was reached on six statements and they were omitted from round 2. In round 2, 32 statements were presented. Based on the results of round 1, 16 statements were adjusted and 16 new statements were formulated. Eight statements were provided with an introduction text or additional remarks based on the comments provided. In round 2, 24 statements were agreed upon leaving 8 with no consensus. In round 3, these 8 remaining statements were adjusted and presented again leading to consensus on another 4, leaving 4 with no consensus. In summary, after three rounds consensus was reached on 34 statements (Table 2) and in 4 cases no consensus was reached (Table 3).

**Table 2:** Statements with consensus reached

Statement per topic	Consensus reached in round	Agree (%)	I dont know (%)
**Testing endurance capacity (8 statements)**			
The effects of endurance capacity training can be adequately evaluated by means of a 6 Minute Walking Test	1	83	0
The 6 min walking test is NOT appropriate to determine the target exercise intensity for endurance capacity training in orthopedic geriatric rehabilitation	2	82	0
For most patients in orthopedic geriatric rehabilitation it is NOT feasible to perform a maximal exercise test (CPET)	2	96	0
For most orthopedic geriatric patients CPET is NOT an appropriate test to evaluate the effects of endurance capacity training	2	96	4
For orthopedic geriatric patients that are not limited by pain and are able to exercise on a (recumbent)cycle ergometer, target exercise intensity (in Watts or HR) for endurance capacity training can be adequately determined by means of an Astrand test	2	82	0
For most orthopedic geriatric patients the Astrand test is NOT an appropriate test to evaluate the effects of endurance capacity training	2	93	4
For most orthopedic geriatric patients the Talk Test is NOT an appropriate test to evaluate the effects of endurance capacity training	2	82	7
For the evaluation of the effect of endurance capacity training in orthopedic geriatric patients the most important outcome is a functional measure, like the performance on a 6 min walking test or a patient specific goal	2	93	0
**Training endurance capacity (9 statements)**			
To achieve an adequate training stimulus, general guidelines for the improvement of endurance (aerobic capacity) should be tailored to individual patients. The ACSM-guidelines are the best available evidence for this purpose: Frequency: at least 3 sessions/week (vigorous intensity), at least 5 sessions/week (moderate intensity) Intensity: Borg 0–10 scale: 5–6 (moderate intensity), 7–8 (vigorous intensity) TIme: 30 to 60 min/session (moderate intensity); 20 to 30 min/session (high intensity)	2	79	11
The ASCM-guideline can be tailored to a patients' needs by starting at a lower intensity and session duration, and progressively increasing intensity and session duration to the recommended guidelines	2	93	4
The ACSM-guidelines can be tailored to a patients' needs by varying in the interplay between frequency, intensity and session duration, for example by providing more frequent, shorter sessions, or by providing longer sessions of lower intensity	2	93	4
A frequency of 3–4 × per week is suitable	1	77	10
Only if the training stimulus is high enough, endurance capacity can improve by means of functional training (walking, sit to stand etc.)	2	86	7
For orthopedic geriatric patients the exercise intensity for endurance capacity training can be adequately monitored by a Modified Borg RPE (scale 0 to 10)	2	93	0
For orthopedic geriatric patients the exercise intensity for endurance capacity training can be monitored the best by a combination of the two aforementioned methods (Modified Borg RPE and ventilation)^#^.	2	75	7
For orthopedic geriatric patients it is possible to monitor the intensity of endurance capacity training WITHOUT SPECIFIC exercise modes, such as walking on a treadmill or cycling on a bicycle ergometer	2	89	7
Endurance capacity training should be continued* after orthopedic geriatric rehabilitation to further increase the effects or to help limit further age-related deterioration *can include daily aerobic physical activities or exercise in different exercise settings e.g. local gym, home based exercise	3	84	4
**Testing muscle strength (5 statements)**			
For patients in orthopedic geriatric rehabilitation for whom improving muscle strength is a goal, a derived 1 RM (e.g. based on 8 or 10RM) test is suitable to determine the target training intensity	2	89	0
For patients in orthopedic geriatric rehabilitation for whom improving muscle strength is a goal, the effects of muscle strength training can be adequately evaluated by a derived 1RM (e.g. based on 8 or 10 RM) test	2	93	0
For patients in orthopedic geriatric rehabilitation for whom improving muscle strength is a goal, the effects of muscle strength training can be adequately evaluated by improvement in functional activities that have a significant strength component	2	96	4
With functional strength training, the desired training intensity can be adequately determined by making functional adjustments to the task (eg by adjusting the height of a seat when getting up from a chair until the exercise can just be performed)	1	87	7
If the desired training intensity cannot be determined (for example due to pain) then it is still useful to do strength training at an intensity that does not cause pain	1	83	3
**Training muscle strength (12 statements)**			
A frequency of 2 × per week is suitable	1	77	3
The number of 2–3 sets per muscle group is suitable	1	87	7
Even if intensity cannot be monitored strength training in orthopedic geriatric rehabilitation is still useful	2	93	0
An intensity of 40–60% of 1RM with at least 15 repetitions per set is adequate to improve local muscular endurance in orthopedic geriatric rehabilitation	2	75	14
The strength training guidelines can be tailored to a patients' needs by varying in the interplay between frequency, intensity and session duration, for example by providing more frequent, shorter sessions, or by providing longer sessions of lower intensity	2	82	4
In orthopedic geriatric rehabilitation in most patients a gradual buildup of strength training intensity is necessary to ensure that the patients' technique is correct before intensity is increased to a recommended level	2	86	0
Specific and controlled strength training using equipment (eg. a leg press or lat pulley) is NOT necessary to improve muscle strength	2	86	0
The effect of functional strength training may be further enhanced by combining it with training on specialized equipment	2	86	11
The intensity of strength training in orthopedic geriatric rehabilitation can be adequately monitored during functional activities (e.g. By the number of reps in a sit to stand exercise)	2	96	0
The maximum number of repetitions attained in a set (and compared to the target number of repetitions) is a feasible and valid measure of monitoring muscle strength training intensity in orthopedic geriatric rehabilitation	3	76	4
The modified BORG RPE scale may be applicable to monitor strength training intensity in orthopedic geriatric patients, without cognitive impairments	3	80	8
Muscle strength improvement can be expected after 6–9 weeks of resistance training and should be continued* after orthopedic geriatric rehabilitation to further increase the effects or to help limit further age-related deterioration *can include daily strength-based physical activities or exercise in different settings e.g. local gym, home-based exercises	3	92	0

#This statement refers to two statements of which one consensus was reached (Modified Borg) and another on which consensus was not reached (i.e. the second statement presented in Table 3: “For orthopedic (…) whole sentences.”

**Table 3:** Statements without consensus reached

Topic	Statement	Agree (%)	I don’t know (%)
Testing endurance capacity	For orthopedic geriatric patients that are able to exercise on a (recumbant)cycle ergometer AND are able to talk during low intensity exercise*, the Talk Test is an adequate tool to determine target exercise intensity (in Watts or HR) for endurance capacity training *i.e. not limited by ventilatory or cognitive functioning	64	4
Training endurance capacity	For orthopedic geriatric patients that are able to exercise on a (recumbant)cycle ergometer AND are able to talk during low intensity exercise*, the exercise intensity for endurance capacity training can be adequately monitored by means of training at a level just below the ventilatory threshold, which means that the patient can just speak in whole sentences *i.e. not limited by ventilatory or cognitive functioning	68	4
Testing muscle strength	For patients in orthopedic geriatric rehabilitation for whom improving muscle strength is indicated as a goal AND resistance exercise is possible, handheld dynamometry is a valuable tool to quantify possible effects of resistance training on local muscle strength, as an addition to more functional evaluation	72	7
Training muscle strength	In patients that are not hindered by pain, an intensity associated with 70–79% of 1RM with a target number of repetitions per set of 7–9 is adequate to improve maximal muscle strength in orthopedic geriatric rehabilitation	60	7

### Qualitative feedback per topic throughout the delphi rounds

To illustrate the evolution of the Delphi study we here present a selection of the qualitative feedback received and the course of the discussion on the various topics. For some quotes we added explanatory text between square brackets.

#### Testing endurance capacity

Of the statements and questions presented in round 1, some were related to specific endurance capacity testing methods such as the cardiopulmonary exercise test (CPET), 6 min walk test (6MWT), Astrand test and the Talk Test. Delphi panelists were pointing out that for orthopedic patients in GR a bicycle ergometer-based test is not a feasible option because of pain. Some questioned the feasibility of a CPET because it requires equipment and trained personnel, and indicated that the population is too frail to undergo a graded exercise test till maximum exertion (maximal exercise test). One Delphi member pointed out that in her practice–for this reason–they don’t even have a bicycle ergometer. Much feedback was provided stating that functional testing should be considered. As one expert put it: “Comment on all the tests so far–they are generally not suitable for frail older people who present with a hip fracture, which is the predominant case load. Therefore, all testing must be applicable and functional for this caseload. Most of the 'research' based cycle ergometer testing is therefore not suitable”. The adjusted statement in round 2 “For the evaluation of the effect of endurance capacity training in orthopedic geriatric patients the most important outcome is a functional measure, like the performance on a 6 min walking test or a patient specific goal” was, therefore, accepted by a large majority (93%). Items that failed to reach consensus in any round were related to the Talk Test to determine target exercise intensity (Table 3). Issues raised in round 2 for instance are: “There are so many older adults who cannot walk and talk or who slow down when speaking. I think this method is difficult for patients in their 80 s.” When the statements were rephrased in such a way that cognition and ventilation are not impaired, the statements were still not accepted (Fig. 2, overview box endurance testing and training).

#### Training endurance capacity

In the first round we presented the FITT characteristics as proposed by the meta-regression analysis of Huang et al. [8] based on 41 RCTs in older adults, but they were found to be too inflexible and too harsh for the orthopedic GR population. As one expert put it: “I am really opposed to a one size fits all approach. The best training program is the one you can get them to do consistently. Starting with 14–15 RPE [rating of perceived exertion on a Borg scale] in the orthopedic population is likely unrealistic. That duration [50–53 weeks] is definitely unrealistic. If you are going to use one prescription as a starting point or guide, use the American College of Sports Medicine (ACSM) guidelines text to derive one. These are well established.” In line with this and other similar comments we adopted the ACSM FITT characteristics in our statements [7, 15], to which the panel agreed to subsequently, along with some supporting statements regarding tailoring of the exercise to the patient (Fig. 2, overview box endurance testing and training). Additionally, it was pointed out that in many patients, activities of daily living can also improve by gradually performing more activities of daily living and that training of aerobic capacity per se should not be a goal in itself. As one expert puts it: “Also with walking with a walker or other specific training (such as transfers) the patient can improve his or her endurance capacity. In some instances even better because it is more functional”. In round 1, a number of potential ways for monitoring exercise intensity for endurance training were proposed. For monitoring intensity during endurance capacity training the Borg scale was evaluated as both feasible and valid. Other options were discarded as being infeasible and or invalid such as percentage of maximum heart rate or oxygen uptake as they would require (an infeasible) maximal test, as well as questionable appropriateness of such parameters in patients who are on cardiac medication. Training on the basis of breathing effort was not perceived as a valid option. In round 2 there was consensus on the statement that intensity of endurance exercise could be measured without using specific controlled exercise modes on a bicycle ergometer or treadmill. In line with this it was agreed by the panel that using the modified Borg scale (Scale 0–10 instead of the traditional 6–20) was the best way to monitor exercise intensity during endurance training. The addition of the observation if the patient can speak comfortably while training alongside the Borg score lowered the number of experts agreeing, meaning that indeed dyspnea is not viewed as key sign when monitoring. This is underscored by the statement on ventilation that was revised for round 3 (Table 3, second statement). It still did not reach consensus and there were given clear reasons for this in the comments. As one expert puts it: “Not reliable enough. Sometimes patients have comorbidities and or cardiac medication that that hampers talking during exercise beforehand”. Monitoring however was deemed to be essential but with a level of pragmatism attached to it.

#### Testing muscle strength

From the statements posed in round 1 and 2 it became clear that a derived 1 repetition maximum (1RM) test, for example based on 8RM, is the best choice to evaluate the effects of muscle strength training as well as to base training intensity on. One expert explicitly stated to not use a standard 1RM test (in which it is determined which high load can be lifted just once): “highly discourage 1RM testing in a population where prevalence of osteoporosis and vertebral fractures likely to be high. Estimated or derived 1 RM may be ok, but formal testing may not make sense at first.” Indeed there was also some skepticism regarding the uses of any form of formal 1RM testing. As one expert formulated: “It is likely that many individuals in the healthy older adult demographic have never engaged in resistance training and I would not advise starting with a 1RM assessment for these individuals. This is also the case within orthopaedic geriatric patients and I believe 1RM testing is completely inappropriate and unnecessary. (…) “. On the other hand, it was also found to be acceptable to base the exercise intensity on the performance on a functional task (as a pragmatic test), provided that it has a significant strength component. The intensity can then be adjusted by e.g. adjusting the height of a seat when getting up from a chair until the exercise can just be performed.

#### Training muscle strength

Regarding FITT characteristics, strength training for 2 sessions per week and 2–3 sets per training was found to be adequate by a majority of panel members. The number of repetitions of 7–9 was approved by many (but did not reach 75% consensus) and may vary according to the specific goal (e.g. may be higher when improvement in local muscular endurance is the goal). A long training period (literature suggested an optimal period as long as 50–53 weeks) was found to not be feasible in orthopedic GR setting. A gradual buildup was also suggested by several experts. The psychosocial aspects of strength training (i.e. improving motivation and adherence) was deemed important, but was out of scope of the current Delphi study. Also it was stressed that any strength training is better than none, as one expert mentioned: “Anything is better than nothing–so even if I cannot measure intensity, I would use strength training in virtually all of my patients”. Regarding monitoring of intensity the panel almost unanimously agreed to monitor intensity by looking at the performance at functional activities (e.g. by the number of repetitions relative to the maximum number, in a sit to stand exercise). One expert is rather pragmatic and says: “you can monitor intensity for any exercise if you standardize your approach to testing. For example, I could ask someone to warm up, then do as many pushups as possible. Let's say they do 8. I could prescribe 3 sets of 6 or 7 to start, and then ask them to increase the number they do every 2 weeks, but keeping 1–2 repetitions in reserve. So their intensity is a consistent percent of their max ability.” Many panelists commented that functional training should be the main focus but could be supported with specific muscle strength training using specialized equipment. “It [specific and controlled strength training using equipment] is not necessary but is useful to have and can provide more variety to a program and potentially make it easier to train specific body parts around a fracture site.” Furthermore, although consensus was reached on one aspect of strength training intensity related to enhancing local muscular endurance, we could not reach consensus on more strenuous intensities. This may have been related on the one hand to the parameters itself “Still seems too arbitrary for these patients who are diverse in their capacity” and on the other hand on phrasing as it was phrased as improvement in maximal strength. “It is more accurate to phrase this, adequate to improve 'muscular strength' rather than 'maximal' muscular strength–because, as noted by several respondents, this is not actually a maximum strength training protocol.. “Some also wished for more flexibility in the parameter range to accommodate to the patient: “I think a statement that better reflects the range of training options would be preferred (…). The intensity you have proposed would obviously work and I don't disagree with that–but we need to recognize that other training plans may be as effective.”

## Discussion

**Fig. 1:**
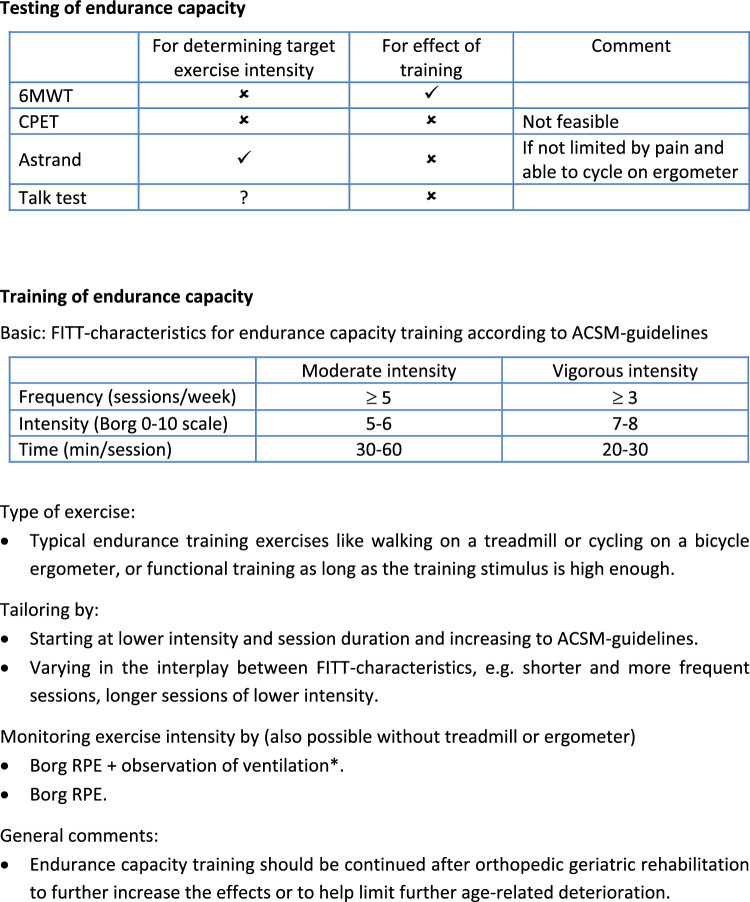
Summary of recommendations of endurance capacity testing and training

**Fig. 2:**
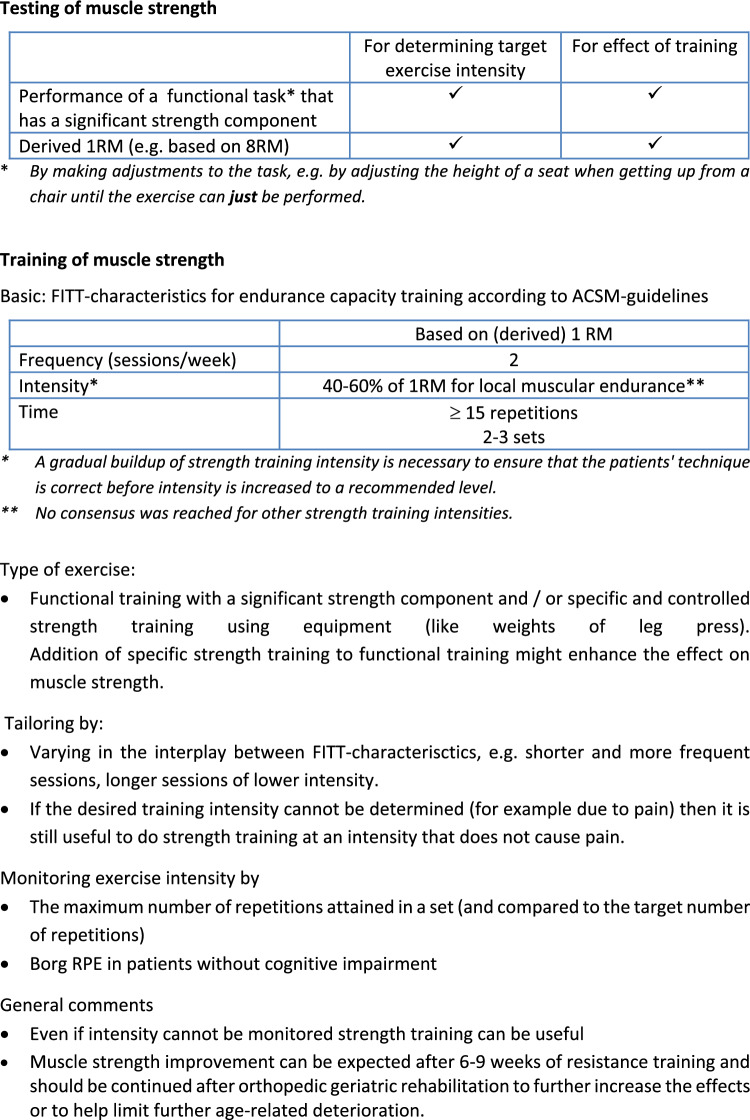
Summary of recommendations regarding muscle strength testing and training

This study provides a first set of practical guidelines for exercise recommendations for older orthopedic patients admitted to GR. We reached consensus on a number of guiding statements regarding exercise testing and training for strength and endurance capacity in these patients. We found that the Delphi panel was very outspoken regarding the use of exercise testing for both strength and endurance. Generally stated, there is a lack of valid and feasible tests, and the panel called for pragmatic approaches, i.e. they found it acceptable to measure exercise intensity through observing performance in functional activities. Although this pragmatic approach may be found to be acceptable in clinical practice, it may be problematic in the context of clinical research where a high degree of validity and accuracy is required to be able to gather evidence on training effectiveness. In addition, we would argue that even in clinical practice it would be beneficial to have reliable and valid outcome measures e.g. to evaluate therapy success and to strive for this whenever possible. There was a general dismay among the Delphi panelists for testing on (bicycle)ergometers because of the patients’ functional limitations and advanced cardiopulmonary exercise testing was found not to be feasible for most patients (reasons provided are e.g. having functional limitations and being frail) and settings (because equipment and skilled personnel is absent). The general tendency of experts was to encourage to just start practicing at the patients’ current fitness level and gradually progress from there even without formal testing procedures, be it endurance or strength training. The panel found that observations of exercise intensity are then best made based on RPE (for endurance capacity training) and by monitoring the maximum number of repetitions attained in a set on a strength task and relating it to the intended number of repetitions. Based on the findings we provided a summary of suggestions for endurance capacity and muscle strength testing and training (Figs. 1 and 2).

Formal endurance capacity testing (e.g. by a CPET) was discouraged by the Delphi panel. And although there are some studies that show that cardiorespiratory fitness can be measured by a CPET in healthy older adults [16], and frailty in itself is not a contraindication for performing a CPET the literature on its use in frail older adults with an impairment is scarce. Thus, there is a need for more research into feasible, valid and reliable ways for testing aerobic capacity in older patients in (orthopedic) GR.

With regard to endurance capacity training the panel endorsed the adherence to ACSM guidelines providing that the load is properly adapted when needed [7, 15]. What is notable however is that in our results endurance exercise training was far less prominently advocated than strength training and that endurance training was expected to occur in the slipstream of performing functional exercises. However our panel stressed that aerobic exercise, in one way or another, should be encouraged after discharge and that striving for ACSM guidelines is a good starting point. One interesting finding was that the Borg score was endorsed in patients that are not hindered by cognitive problems for muscle strength and endurance capacity training. In line with this, Bok et al. [17] argue in their recent review that subjective ratings of exercise intensity for endurance training may be as effective as intensities based on prior formal graded (maximal) testing. However most of the studies are performed in (young) adults [17].

When we compare the consensus results of muscle strength testing and training to existing recommendations of ACSM [7] and of the National Strength and Conditioning Association [5] and of Izqueirdo et al. [6], we find that there is no explicit mention of exercise testing to determine or monitor exercise intensity, but rather an implicit reference to 1RM measurement for strength and intensity monitoring during training for endurance exercise (no a priori testing). There is indeed hardly any literature on exercise testing methods in frail older patients let alone orthopedic GR patients. For testing muscle strength, performance in a functional task (e.g. standing up from a chair for strength) as well as derived 1RM testing is suggested by our Delphi panel. This functional-based method is in some way related to a derived 1RM testing method. With regard to training, the parameters stated for strength training correspond with our findings regarding the number of training sessions and sets. The number of repetitions and intensity stated in the position statement are however somewhat different, with a lower number of repetitions and a broad intensity range starting at 20% and working up to 80% of 1RM. We did not reach consensus on intensities at the higher end of the spectrum (e.g. 70–80% of 1RM) potentially because of issues with phrasing and small range of repetitions. From the data it was however clear that the common notion of 80% 1RM for 8–12 repetitions was not actively promoted in the feedback, suggesting that such an approach may not be very common in orthopedic GR patients. In the literature there is some evidence that functional task exercise is more effective in improving daily functioning than (non-functional) resistance exercise [18]. In another study in the oldest old, strength exercises did improve leg muscle strength but failed to translate to improved physical functioning as measured by 4-step stair test and timed up and go test [19]. Apart from this, a study in frail recently hospitalized patients showed that a high intensity resistance exercise program (i.e. 60–80% of 1RM) led to more musculoskeletal injuries [20], which shows that some caution may be needed for higher intensity programs. Also the short time that patients are in rehabilitation wards may contribute to a focus on functional activities such that patients can go home and perform basic activities of daily living themselves. However, returning home should not be the end of the therapy. The panelists agree that -ideally- there should be a long term follow-up of both strength and endurance training activities integrated into the daily lives as much as possible. Currently it is unknown what the best way is –both from a health services and exercise physiology perspective to support orthopedic GR patients in the long run when they have returned home.

This study shows that with regard to orthopedic GR there are several remaining uncertainties that should be subject of further study. First, regarding exercise testing, there is a need of evidence on the several methods currently used in practice as well as the ones that are underutilized (e.g. testing protocols for endurance capacity). It is of interest to compare subjective (i.e. RPE based) with objective (i.e. derived 1RM based) strength training prescriptions with regard to efficacy in this population (or in frail patients in general) to strengthen the knowledge base on this issue. With regard to muscle strength training, the optimal (yet feasible) training intensities for orthopedic GR patients are still rather unknown and may also be related to the functional goals one wants to attain. It may be worthwhile examining which type of muscle strength (e.g. maximal strength, strength endurance, power) is required for different functional activities in orthopedic GR and what the optimal parameters are for each of those. Furthermore, alternative strength training methods that may enhance strength gains without the need for heavy loading such as resistance training with vascular occlusion [21] may be particularly promising in orthopedic GR patients where the load-taking capacity is often reduced. Lastly, for both strength and endurance training it was stressed that there should be a long term follow up to ensure full benefits and prevent future health issues. How to organize this transitional care to accommodate this goal in a sustainable and cost efficient manner is an important question for further research. Also, more research is needed on the impact of varying levels of frailty and cognition in this population and how to accommodate testing and training procedures to these measures. Our recent literature review about endurance training in patients with different types of frailty may provide a starting point for this [10].

A strength of the study is that we included a diverse group of experts consisting of researchers and clinicians involved in the care for patients in orthopedic GR, which means that the statements are backed by practical and scientific views. Another strength is the relatively high compliance of participants across the different Delphi rounds, which limits the potential for bias from selective dropout. Limitations of the study include the relatively small number of experts and an overrepresentation of Dutch physiotherapists. We had a fixed number of three Delphi rounds and some statements in the first round may have been too explorative and other statements may have been rather provocative to reach a positive consensus on. Although physical therapists and clinical exercise physiologists can use this as a guide when training their frail patients in orthopedic GR. It is by no means a definite guideline but rather a starting point that may be amended in the coming years as more evidence will be becoming available. Lastly, the current findings are restricted to the views of experts. The patients’ and carers’ perspectives were investigated in another study which is submitted elsewhere.

In conclusion, we have reached agreement on multiple strength and endurance testing and training characteristics for frail older adults in orthopedic GR. Generally stated, methods used for testing and training should be pragmatic in nature and a functional approach to exercise training is preferred. For endurance training existing guidelines of the American College of Sports Medicine can be strived for but adapted as needed and for muscle strength training only lower intensities are agreed upon. There are several remaining uncertainties that need further study, such as the optimal muscle strength training intensity and optimal strategies for long term support of these patients at home or in the community.

### Supplementary Information

Below is the link to the electronic supplementary material.

Supplementary file1 (PDF 215 KB)

Supplementary file2 (PDF 561 KB)

## Data Availability

The participants of this study did not give written consent for their data to be shared publicly, so supporting data is not available.
