# Supplementary material for: Exercise testing and training in frail older adults with an orthopedic impairment participating in a geriatric rehabilitation program: an international Delphi study
Source: Eur Geriatr Med. 2023 Jul 4;14(5):985–97. doi: 10.1007/s41999-023-00819-5 (PMC10587272; doi:10.1007/s41999-023-00819-5)
Supplement: Supplementary file 1 — Supplementary file1 (PDF 215 KB) [file 41999_2023_819_MOESM1_ESM.pdf]

Online Resource 1: Background information of the Delphi study

Background information pertaining to:

Expert consensus on physical testing and training in elderly patients rehabilitating after orthopedic surgery: An International Delphi Study

This document contains background information and definitions and descriptions that are important to properly answer the statements and questions in the Delphi study. Please read this carefully and use it when completing the Delphi.

Introduction and demarcation of target group

This Delphi study aims to reach consensus on aspects of physical testing and training parameters in strength and endurance training in elderly patients rehabilitating after **orthopedic surgery**.

There is still limited evidence in the literature as to what appropriate and effective physical tests and training parameters for endurance and strength training are for this population. Little is known, especially about training intensity.

The statements (and some questions) presented in this Delphi study are presented for the situation where **strength and / or endurance training is part of the rehabilitation pathway to achieve the rehabilitation goals**. The question is not whether strength and endurance training **is** indicated, but if it **is** indicated, then how should it be designed.

Although rehabilitation of these patients is always multidisciplinary in nature, this research focuses strictly on the aspect of **physical training** as provided by physiotherapists, whether or not supported by exercise physiologists.

Definitions and descriptions

Here are some descriptions of endurance and strength tests.

Endurance capacity testing

Below are some exercise tests (the list is not exhaustive) that are used to measure endurance capacity.

Maximal Exercise Test: Cardiopulmonary Exercise Test (CPET)

This is a symptom-limited test of approximately 8-12 minutes, in which the patient performs an effort on a bicycle ergometer or treadmill that is gradually increased (usually 10 to 20 W per minute) until the patient is unable to continue despite verbal encouragement. This test is also referred to as the cardiopulmonary exercise test (CPET). Mainly, but not exclusively, for prognostic and diagnostic purposes, the test is therefore usually combined measurements of the ECG, blood pressure and heart rate (HR). Also, breathing gas analyses are performed to determine relevant exercise physiological parameters, including maximum aerobic capacity (VO2 peak) and the ventilation threshold(s).

Sub-maximal exercise test: Astrand Rhyming test

The Astrand test is a submaximal exercise test that is usually performed on a calibrated bicycle ergometer on which you cycle at a specific load for 6 minutes. The heart rate should be between 130 and 170 beats per minute at the end of these 6 minutes. Subsequently, an estimate of the VO2max can be made by means of the so-called Astrand Rhyming nomogram, using the attained power and the heart rate.

Six-minute walking test (6MWT)

The six-minute walk test is a test in which the person is asked to walk as long as possible (without running) in 6 minutes on a marked trail, ranging in literature from 8 to 50 meters. In addition, a standardized encouragement is given every minute. The most important outcome of the test is the total distance covered in meters. Requirements are a stopwatch and tape measure. Optionally, a heart rate monitor, BORG scale, or pulse oximeter can be used to record the physiological response of the test.

Sub-maximal exercise test: Talk test

The Talk test is an alternative exercise test in which an ascending exercise protocol is performed on a treadmill or bicycle ergometer. The load steps are each 2 minutes with the load increasing 10 to 20 W per minute. The patient is asked to say a standard text every last 30 seconds and then to indicate whether they could still talk comfortably. Answer options are yes, doubt (yes, but…) or no. The load step at which the patient voices the doubt is then used as an estimator for the ventilatory threshold.

Testing muscle strength

1 Reptition Maximum (1RM) test.

Muscle strength is usually tested by means of the 1 Reptition Maximum (1RM) method and usually takes place on a strength training machine (eg. leg press, lat pull-down). The test means that, according to a specific protocol, it is determined within a few measurements how much weight the patient can move a maximum of 1 time with the relevant muscle group. Usually the 1RM is ultimately derived from a sub-maximal load of eg. 8 repetitions and the 1RM is estimated.

Physical training

FITT Factors

Physical training is based on the principle of progressive overload: the load during training is above a minimum (threshold) value, and increases as endurance increases. The load is determined by the Frequency (how often per week), the Intensity (how heavy) and the Time (how long in a row). Together with the Type of activity, these form the FITT characteristics of training.

Endurance training

Endurance training refers to longer physical activity involving large muscle groups and significantly increasing metabolism. Examples are activities such as cycling, walking or running, swimming, ball game exercises

Muscle strength training

- *Functional strength training*

When we refer to the statements about functional strength training, we mean exercises in which a functional act is performed that contains a strength component. Examples include: 1 or 2-legged squats, getting on and off an elevation, lunges, climbing stairs, getting up from a chair, etc.

- *Strength training with weights or equipment*

With this form of strength training we mean exercises that are performed with the help of either a machine or with dumbbells or other weights, whereby a muscle group is addressed separately. Examples of exercises on a machine are leg extension, leg press, leg flexion, calf raises, lat pulldown. Examples of exercises with weights are for example biceps curl, bench press, bent over rowing etc.
