# Supplementary material for: Exercise testing and training in frail older adults with an orthopedic impairment participating in a geriatric rehabilitation program: an international Delphi study
Source: Eur Geriatr Med. 2023 Jul 4;14(5):985–97. doi: 10.1007/s41999-023-00819-5 (PMC10587272; doi:10.1007/s41999-023-00819-5)
Supplement: Supplementary file 2 — Supplementary file2 (PDF 561 KB) [file 41999_2023_819_MOESM2_ESM.pdf]

Online Resource 2. Questions and statements of the three Delphi rounds

Round 1 (Pages 1 - 15)

Round 2 (Pages 16 - 23)

Round 3 (Pages 24 - 26)

Question	Row	Choice / Column	Values
**Sociodemographics**			
What is your country of residence?			
What is your age?			
What is your sex?			{1:Male}, {2:Female}, {3:Other}
What is your main profession?			{1:Physical therapist}, {2:Researcher}, {3:MD}, {4:Other}
How many years of experience do you have in this profession?			
How many years (scientific or clincal) experience do you have regarding physcal testing and/or training of elderly patients in general?			
How many years (scientific or clincal) experience do you have regarding physical testing and/or training of elderly patients who are rehabilating from an orthopedic procedure such as a hip fracture?			
**Part 1: Testing endurance capacity**			
1. Each geriatric patient must undergo a maximal exercise test (CPET) prior to any exercise program to ensure patient safety during physical training.			{1:Strongly disagree}, {2:Disagree}, {3:Agree}, {4:Strongly agree}, {5:I don't know}

If desired, you can make an explanatory statement here:			
2. Each patient for whom improvement of endurance capacity is a goal should undergo a maximal exercise test (CPET) prior to an exercise program to determine the target exercise intensity for endurance capacity training.			{1:Strongly disagree}, {2:Disagree}, {3:Agree}, {4:Strongly agree}, {5:I don't know}
If desired, you can make an explanatory statement here.			
3. The target exercise intensity for endurance capacity training can be adequately determined by means of an Astrand test.			{1:Strongly disagree}, {2:Disagree}, {3:Agree}, {4:Strongly agree}, {5:I don't know}
If desired, you can make an explanatory statement here.			
4. The target exercise intensity for endurance capacity training can be adequately determined by means of a 6 minute walking test.			{1:Strongly disagree}, {2:Disagree}, {3:Agree}, {4:Strongly agree}, {5:I don't know}
If desired, you can make an explanatory statement here.			
5. The target exercise intensity for endurance capacity training can be adequately determined by means of a Talk Test.			{1:Strongly disagree}, {2:Disagree}, {3:Agree}, {4:Strongly agree}, {5:I don't know}
If desired, you can make an explanatory statement here.			
6. The effects of the endurance capacity training can be adequately evaluated by means of a maximal exercise test (CPET).			{1:Strongly disagree}, {2:Disagree}, {3:Agree}, {4:Strongly agree}, {5:I don't know}

If desired, you can make an explanatory statement here.			
7. The effects of endurance capacity training can be adequately evaluated by means of an Astrand test.			{1:Strongly disagree}, {2:Disagree}, {3:Agree}, {4:Strongly agree}, {5:I don't know}
If desired, you can make an explanatory statement here.			
8. The effects of endurance capacity training can be adequately evaluated by means of a 6 Minute Walking Test.			{1:Strongly disagree}, {2:Disagree}, {3:Agree}, {4:Strongly agree}, {5:I don't know}
If desired, you can make an explanatory statement here.			
9. The effects of endurance capacity training can be adequately evaluated by means of a talk test.			{1:Strongly disagree}, {2:Disagree}, {3:Agree}, {4:Strongly agree}, {5:I don't know}
If desired, you can make an explanatory statement here.			
10. Which (exercise) test is at least as suitable as the CPET to:<br>- Determine the safety of endurance capacity training?			
11. Which (exercise) test is at least as suitable as the CPET, Astrand test, 6 Minute Walk Test or Talk test to<br>- Determine the target exercise intensity for endurance capacity training?			
12. Which (exercise) test is at least as suitable as the CPET, Astrand test, 6 Minute Walk Test or Talk test<br>- Evaluate the effect of endurance capacity training?			

13. Do you feel that something is missing related to TESTING endurance capacity in the elderly in orthopedic geriatric rehabilitation? If yes, please explain.			
**Part 2: TRAINING endurance capacity**			
14. For each of the methods below, indicate whether they are suitable for monitoring exercise intensity during endurance capacity training in orthopedic geriatric rehabilitation. Score each method on feasibility (is it possible?) and validity (do you measure what you intend to measure?).	Percentage of the maximum heart rate	feasible	{0:0}, {1:1}
14. For each of the methods below, indicate whether they are suitable for monitoring exercise intensity during endurance capacity training in orthopedic geriatric rehabilitation. Score each method on feasibility (is it possible?) and validity (do you measure what you intend to measure?).	Percentage of the maximum heart rate	valid	{0:0}, {1:1}
14. For each of the methods below, indicate whether they are suitable for monitoring exercise intensity during endurance capacity training in orthopedic geriatric rehabilitation. Score each method on feasibility (is it possible?) and validity (do	Percentage of the maximum heart rate	I don't know	{0:0}, {1:1}

you measure what you intend to measure?).			
14. For each of the methods below, indicate whether they are suitable for monitoring exercise intensity during endurance capacity training in orthopedic geriatric rehabilitation. Score each method on feasibility (is it possible?) and validity (do you measure what you intend to measure?).	Percentage of the heart rate reserve	feasible	{0:0}, {1:1}
14. For each of the methods below, indicate whether they are suitable for monitoring exercise intensity during endurance capacity training in orthopedic geriatric rehabilitation. Score each method on feasibility (is it possible?) and validity (do you measure what you intend to measure?).	Percentage of the heart rate reserve	valid	{0:0}, {1:1}
14. For each of the methods below, indicate whether they are suitable for monitoring exercise intensity during endurance capacity training in orthopedic geriatric rehabilitation. Score each method on feasibility (is it possible?) and validity (do you measure what you intend to measure?).	Percentage of the heart rate reserve	I don't know	{0:0}, {1:1}

14. For each of the methods below, indicate whether they are suitable for monitoring exercise intensity during endurance capacity training in orthopedic geriatric rehabilitation. Score each method on feasibility (is it possible?) and validity (do you measure what you intend to measure?).	Percentage of peak oxygen uptake	feasible	{0:0}, {1:1}
14. For each of the methods below, indicate whether they are suitable for monitoring exercise intensity during endurance capacity training in orthopedic geriatric rehabilitation. Score each method on feasibility (is it possible?) and validity (do you measure what you intend to measure?).	Percentage of peak oxygen uptake	valid	{0:0}, {1:1}
14. For each of the methods below, indicate whether they are suitable for monitoring exercise intensity during endurance capacity training in orthopedic geriatric rehabilitation. Score each method on feasibility (is it possible?) and validity (do you measure what you intend to measure?).	Percentage of peak oxygen uptake	I don't know	{0:0}, {1:1}
14. For each of the methods below, indicate whether they are suitable for monitoring exercise intensity during endurance capacity training in orthopedic geriatric rehabilitation. Score each method on feasibility (is it possible?) and validity (do you measure what you intend to measure?).	Training on the basis of breathing difficulty: can the patient still talk without difficulty?	feasible	{0:0}, {1:1}

14. For each of the methods below, indicate whether they are suitable for monitoring exercise intensity during endurance capacity training in orthopedic geriatric rehabilitation. Score each method on feasibility (is it possible?) and validity (do you measure what you intend to measure?).	Training on the basis of breathing difficulty: can the patient still talk without difficulty?	valid	{0:0}, {1:1}
14. For each of the methods below, indicate whether they are suitable for monitoring exercise intensity during endurance capacity training in orthopedic geriatric rehabilitation. Score each method on feasibility (is it possible?) and validity (do you measure what you intend to measure?).	Training on the basis of breathing difficulty: can the patient still talk without difficulty?	I don't know	{0:0}, {1:1}
14. For each of the methods below, indicate whether they are suitable for monitoring exercise intensity during endurance capacity training in orthopedic geriatric rehabilitation. Score each method on feasibility (is it possible?) and validity (do you measure what you intend to measure?).	BORG RPE scale (rating of perceived exertion)	feasible	{0:0}, {1:1}
14. For each of the methods below, indicate whether they are suitable for monitoring exercise intensity during endurance capacity training in orthopedic geriatric rehabilitation. Score each method on feasibility (is it possible?) and validity (do you measure what you intend to measure?).	BORG RPE scale (rating of perceived exertion)	valid	{0:0}, {1:1}

14. For each of the methods below, indicate whether they are suitable for monitoring exercise intensity during endurance capacity training in orthopedic geriatric rehabilitation. Score each method on feasibility (is it possible?) and validity (do you measure what you intend to measure?).	BORG RPE scale (rating of perceived exertion)	I don't know	{0:0}, {1:1}
I have another suggestion, namely….			
If desired, you can make an explanatory statement here.			
15. Training of endurance capacity is only useful if the intensity can be monitored.			{1:Strongly disagree}, {2:Disagree}, {3:Agree}, {4:Strongly agree}, {5:I don't know}
If desired, you can make an explanatory statement here.			
16. Only with SPECIFIC endurance capacity training, such as walking on a treadmill or cycling on a bicycle ergometer, the intensity can be monitored.			{1:Strongly disagree}, {2:Disagree}, {3:Agree}, {4:Strongly agree}, {5:I don't know}
If desired, you can make an explanatory statement here.			
17. Specific and controlled endurance capacity training, such as walking on a treadmill or cycling on a bicycle ergometer, is necessary to improve endurance.			{1:Strongly disagree}, {2:Disagree}, {3:Agree}, {4:Strongly agree}, {5:I don't know}
If desired, you can make an explanatory statement here.			
18. The FITT characteristics as described above are suitable for endurance capacity training in the orthopedic geriatric rehabilitation:	A frequency of 3-4x per week is suitable.		{1:Strongly disagree}, {2:Disagree}, {3:Agree}, {4:Strongly agree}, {5:I don't know}

18. The FITT characteristics as described above are suitable for endurance capacity training in the orthopedic geriatric rehabilitation:	An intensity is 66-73% of heart rate reserve (HRR) or a Borg RPE score 14.5 to 15.5 (on a scale of 6 to 20) is suitable.		{1:Strongly disagree}, {2:Disagree}, {3:Agree}, {4:Strongly agree}, {5:I don't know}
18. The FITT characteristics as described above are suitable for endurance capacity training in the orthopedic geriatric rehabilitation:	A duration per workout is 40-50 minutes is suitable.		{1:Strongly disagree}, {2:Disagree}, {3:Agree}, {4:Strongly agree}, {5:I don't know}
18. The FITT characteristics as described above are suitable for endurance capacity training in the orthopedic geriatric rehabilitation:	A total program duration of 30-40 weeks is suitable.		{1:Strongly disagree}, {2:Disagree}, {3:Agree}, {4:Strongly agree}, {5:I don't know}
If desired, you can make an explanatory statement here.			
19. In orthopedic geriatric rehabilitation it is even better to train endurance capacity every day.			{1:Strongly disagree}, {2:Disagree}, {3:Agree}, {4:Strongly agree}, {5:I don't know}
If desired, you can make an explanatory statement here.			
20. Do you feel that something is missing related to TRAINING endurance capacity in the elderly in orthopedic geriatric rehabilitation? If yes, please explain:			
**Part 3: TESTING muscle strength**			
21. Each patient, for whom improving muscle strength is a goal, must undergo a (derrived) 1RM test prior to a training			{1:Strongly disagree}, {2:Disagree}, {3:Agree}, {4:Strongly agree}, {5:I don't know}

program to determine the desired training intensity for strength training.			
If desired, you can make an explanatory statement here.			
22. For each patient for whom improving muscle strength is a goal, a (derived) 1RM test should be used to evaluate the effects of the strength training.			{1:Strongly disagree}, {2:Disagree}, {3:Agree}, {4:Strongly agree}, {5:I don't know}
If desired, you can make an explanatory statement here.			
23. With functional strength training, the desired training intensity can be adequately determined by making functional adjustments to the task (eg by adjusting the height of a seat when getting up from a chair until the exercise can just be performed).			{1:Strongly disagree}, {2:Disagree}, {3:Agree}, {4:Strongly agree}, {5:I don't know}
If desired, you can make an explanatory statement here.			
24. If the desired training intensity cannot be determined (for example due to pain) then it is still useful to do strength training at an intensity that does not cause pain.			{1:Strongly disagree}, {2:Disagree}, {3:Agree}, {4:Strongly agree}, {5:I don't know}
If desired, you can make an explanatory statement here.			
25. Which (strength) test is at least as suitable as the (derived) 1RM test to:<br>- determine the desired training intensity for strength training?			

26. Which (strength) test is at least as suitable as the (derived) 1RM test to:<br>- evaluate the effect of strength training?			
27. Do you feel anyting is missing related to TESTING muscle strength in the elderly in orthopedic geriatric rehabilitation? If yes, please explain.			
28. For each of the methods below, indicate whether they are suitable for monitoring the intensity during muscle strength training in geriatric rehabilitation. Score each method on feasibility (is it possible?) AND validity (do you measure what you would like to measure). Check the boxes if you think the method is feasible and valid or leave them open if you think they are not.	Weight as a percentage of the (in) directly determined 1RM	Feasible	{0:0}, {1:1}
28. For each of the methods below, indicate whether they are suitable for monitoring the intensity during muscle strength training in geriatric rehabilitation. Score each method on feasibility (is it possible?) AND validity (do you measure what you would like to measure). Check the boxes if you think the method is feasible and valid or leave them open if you think they are not.	Weight as a percentage of the (in) directly determined 1RM	Valid	{0:0}, {1:1}

28. For each of the methods below, indicate whether they are suitable for monitoring the intensity during muscle strength training in geriatric rehabilitation. Score each method on feasibility (is it possible?) AND validity (do you measure what you would like to measure). Check the boxes if you think the method is feasible and valid or leave them open if you think they are not.	Weight as a percentage of the (in) directly determined 1RM	I don't know	{0:0}, {1:1}
28. For each of the methods below, indicate whether they are suitable for monitoring the intensity during muscle strength training in geriatric rehabilitation. Score each method on feasibility (is it possible?) AND validity (do you measure what you would like to measure). Check the boxes if you think the method is feasible and valid or leave them open if you think they are not.	Number of repetitions per set	Feasible	{0:0}, {1:1}
28. For each of the methods below, indicate whether they are suitable for monitoring the intensity during muscle strength training in geriatric rehabilitation. Score each method on feasibility (is it possible?) AND validity (do you measure what you would like to measure). Check the boxes if you think the method is feasible and valid or leave them open if you think they are not.	Number of repetitions per set	Valid	{0:0}, {1:1}

28. For each of the methods below, indicate whether they are suitable for monitoring the intensity during muscle strength training in geriatric rehabilitation. Score each method on feasibility (is it possible?) AND validity (do you measure what you would like to measure). Check the boxes if you think the method is feasible and valid or leave them open if you think they are not.	Number of repetitions per set	I don't know	{0:0}, {1:1}
28. For each of the methods below, indicate whether they are suitable for monitoring the intensity during muscle strength training in geriatric rehabilitation. Score each method on feasibility (is it possible?) AND validity (do you measure what you would like to measure). Check the boxes if you think the method is feasible and valid or leave them open if you think they are not.	BORG RPE scale (rating of perceived exertion)	Feasible	{0:0}, {1:1}
28. For each of the methods below, indicate whether they are suitable for monitoring the intensity during muscle strength training in geriatric rehabilitation. Score each method on feasibility (is it possible?) AND validity (do you measure what you would like to measure). Check the boxes if you think the method is feasible and valid or leave them open if you think they are not.	BORG RPE scale (rating of perceived exertion)	Valid	{0:0}, {1:1}

28. For each of the methods below, indicate whether they are suitable for monitoring the intensity during muscle strength training in geriatric rehabilitation. Score each method on feasibility (is it possible?) AND validity (do you measure what you would like to measure). Check the boxes if you think the method is feasible and valid or leave them open if you think they are not.	BORG RPE scale (rating of perceived exertion)	I don't know	{0:0}, {1:1}
I have another suggestion, namely ...			
If desired, you can make an explanatory statement here.			
29. Strength training is only useful if the intensity can be monitored.			{1:Strongly disagree}, {2:Disagree}, {3:Agree}, {4:Strongly agree}, {5:I don't know}
If desired, you can make an explanatory statement here.			
30. Only with specific strength training where equipment is used (eg. a leg press or lat pulley) the intensity can be monitored.			{1:Strongly disagree}, {2:Disagree}, {3:Agree}, {4:Strongly agree}, {5:I don't know}
If desired, you can make an explanatory statement here.			
31. Specific and controlled strength training using equipment (eg. a leg press or lat pulley) is necessary to improve muscle strength.			{1:Strongly disagree}, {2:Disagree}, {3:Agree}, {4:Strongly agree}, {5:I don't know}
If desired, you can make an explanatory statement here.			
32. Training with equipment is more effective for increasing muscle strength than functional strength training.			{1:Strongly disagree}, {2:Disagree}, {3:Agree}, {4:Strongly agree}, {5:I don't know}

If desired, you can make an explanatory statement here.			
33. This guideline for strength training in healthy elderly people also applies to patients within the orthopedic geriatric rehabilitation.	A frequency of 2 x per week is suitable.		{1:Strongly disagree}, {2:Disagree}, {3:Agree}, {4:Strongly agree}, {5:I don't know}
33. This guideline for strength training in healthy elderly people also applies to patients within the orthopedic geriatric rehabilitation.	The number of 2-3 sets per muscle group is suitable.		{1:Strongly disagree}, {2:Disagree}, {3:Agree}, {4:Strongly agree}, {5:I don't know}
33. This guideline for strength training in healthy elderly people also applies to patients within the orthopedic geriatric rehabilitation.	An appropriate number of reps per set of 7-9 is suitable.		{1:Strongly disagree}, {2:Disagree}, {3:Agree}, {4:Strongly agree}, {5:I don't know}
33. This guideline for strength training in healthy elderly people also applies to patients within the orthopedic geriatric rehabilitation.	A total training duration of 50 -53 weeks is suitable.		{1:Strongly disagree}, {2:Disagree}, {3:Agree}, {4:Strongly agree}, {5:I don't know}
If desired, you can make an explanatory statement here.			
34. Do you feel something is missing with regard to the training of muscle strength in the elderly in orthopedic geriatric rehabilitation? If yes, please explain.			
35. Are there any final remarks you wish to make ?			

Round 2

Question	Values
**Part 1: Testing endurance capacity**	
Adjusted statement: For most patients in orthopedic geriatric rehabilitation it is NOT feasible to perform a maximal exercise tes (CPET).	{1:Strongy disagree}, {2:Disagree}, {3:Agree}, {4:Strongly agree}, {5:I don't know}
If desired, you can make an explanatory statement here:	
Adjusted statement: For orthopedic geriatric patients that are not limited by pain and are able to exercise on a (recumbant)cycle ergometer, target exercise intensity (in Watts or HR) for endurance capacity training can be adequately determined by means of an Astrand test.	{1:Strongy disagree}, {2:Disagree}, {3:Agree}, {4:Strongly agree}, {5:I don't know}
If desired, you can make an explanatory statement here:	
Adjusted statement: The 6 minute walking test is NOT appropriate to determine the target exercise intensity for endurance capacity training in orthopedic geriatric rehabilitation.	{1:Strongy disagree}, {2:Disagree}, {3:Agree}, {4:Strongly agree}, {5:I don't know}
If desired, you can make an explanatory statement here:	
Adjusted statement: For orthopedic geriatric patients that are not limited by pain and are able to exercise on a (recumbant)cycle ergometer, target exercise intensity (in Watts or HR) for endurance capacity training can be adequately determined by means of an Talk Test.	{1:Strongy disagree}, {2:Disagree}, {3:Agree}, {4:Strongly agree}, {5:I don't know}
If desired, you can make an explanatory statement here:	
Adjusted statement: For most orthopedic geriatric patients CPET is NOT an appropriate test to evaluate the effects of endurance capactiy training.	{1:Strongy disagree}, {2:Disagree}, {3:Agree}, {4:Strongly agree}, {5:I don't know}

If desired, you can make an explanatory statement here:	
Adjusted statement: For most orthopedic geriatric patients the Astrand test is NOT an appropriate test to evaluate the effects of endurance capacitiy training.	{1:Strongy disagree}, {2:Disagree}, {3:Agree}, {4:Strongly agree}, {5:I don't know}
If desired, you can make an explanatory statement here:	
Adjusted statement: For most orthopedic geriatric patients the Talk Test is NOT an appropriate test to evaluate the effects of endurance capacity training.	{1:Strongy disagree}, {2:Disagree}, {3:Agree}, {4:Strongly agree}, {5:I don't know}
If desired, you can make an explanatory statement here:	
New statement: For the evaluation of the effect of endurance capacity training in orthopedic geriatric patients the most important outcome is a functional measure, like the performance on a 6 minute walking test or a patient specific goal.	{1:Strongy disagree}, {2:Disagree}, {3:Agree}, {4:Strongly agree}, {5:I don't know}
If desired, you can make an explanatory statement here:	
Do you want to make a summary remark regarding TESTING endurance capacity in orthopedic geriatric rehabiliation?	
**Part 2: TRAINING endurance capacity**	

New statement: For orthopedic geriatric patients the exercise intensity for endurance capacity training can be adequately monitored by a Modified Borg RPE (scale 1 to 10).	{1:Strongy disagree}, {2:Disagree}, {3:Agree}, {4:Strongly agree}, {5:I don't know}
If desired, you can make an explanatory statement here:	
New statement: For orthopedic geriatric patients the exercise intensity for endurance capacity training can be adequately monitored by means of training at a level just below the ventilatory threshold, which means that the patient can just speak in whole sentences comfortably.	{1:Strongy disagree}, {2:Disagree}, {3:Agree}, {4:Strongly agree}, {5:I don't know}
If desired, you can make an explanatory statement here:	
New statement: For orthopedic geriatric patients the exercise intensity for endurance capacity training can be monitored the best by a combination of the two aforementioned methods (Modified Borg RPE and ventilation).	{1:Strongy disagree}, {2:Disagree}, {3:Agree}, {4:Strongly agree}, {5:I don't know}
If desired, you can make an explanatory statement here:	
Adjusted statement: For orthopedic geriatric patients it is possible to monitor the intensity of endurance capacity training WITHOUT SPECIFIC exercise modes, such as walking on a treadmill or cycling on a bicycle ergometer.	{1:Strongy disagree}, {2:Disagree}, {3:Agree}, {4:Strongly agree}, {5:I don't know}
If desired, you can make an explanatory statement here:	
Adjusted statement: Only if the training stimulus is high enough, endurance capacity can improve by means of functional training (walking, sit to stand etc).	{1:Strongy disagree}, {2:Disagree}, {3:Agree}, {4:Strongly agree}, {5:I don't know}
If desired, you can make an explanatory statement here:	
New statement: To achieve an adequate training stimulus, general guidelines for the improvement of endurance (aerobic capacity) should be tailored to individual patients. The ACSM-guidelines are the best available evidence for this purpose:<br>- Frequency: at least 3 sessions/week (vigorous intensity), at least 5 sessions/week (moderate intensity)	{1:Strongy disagree}, {2:Disagree}, {3:Agree}, {4:Strongly agree}, {5:I don't know}

- Intensity: Borg 0-10 scale: 5-6 (moderate intensity), 7-8 (vigorous intensity)<br>- TIme: 30 to 60 min/session (moderate intensity) ; 20 to 30 min/session (high intensity)	
If desired, you can make an explanatory statement here:	
New statement: The ASCM-guideline can be tailored to a patients' needs by starting at a lower intensity and session duration, and progressively increasing intensity and session duraction to the recommended guidelines.	{1:Strongy disagree}, {2:Disagree}, {3:Agree}, {4:Strongly agree}, {5:I don't know}
If desired, you can make an explanatory statement here:	
New statement: The ACSM-guidelines can be tailored to a patients' needs by varying in the interplay between frequency, intensity and session duration, for example by providing more frequent, shorter sessions, or by providing longer sessions of lower intensity.	{1:Strongy disagree}, {2:Disagree}, {3:Agree}, {4:Strongly agree}, {5:I don't know}
If desired, you can make an explanatory statement here:	
New statement: In order to achieve optimal endurance capactiy, the training program should continue after orthopedic geriatric rehabilitation, until a total training duration of at least 30 weeks.	{1:Strongy disagree}, {2:Disagree}, {3:Agree}, {4:Strongly agree}, {5:I don't know}
If desired, you can make an explanatory statement here:	
Do you want to make a summary remark regarding TRAINING endurance capacity orthopedic geriatric rehabiliation?	

**Part 3: Testing muscle strength**	
Adjusted statement: For patients in orthopedic geriatric rehabilitation for whom improving muscle strength is a goal, a derived 1 RM (e.g. based on 8 or 10RM) test is suitable to determine the target training intensity.<br><br>(N.B. The majortity (>75%) of the respondents already agreed on the following statement : "With functional strength training, the desired training intensity can be adequately determined by making functional adjustments to the task (eg by adjusting the height of a seat when getting up from a chair until the exercise can just be performed)".)	{1:Strongy disagree}, {2:Disagree}, {3:Agree}, {4:Strongly agree}, {5:I don't know}
If desired, you can make an explanatory statement here:	
New statement: For patients in orthopedic geriatric rehabilitation for whom improving muscle strength is a goal, the effects of muscle strength training can be adequately evaluated by improvement in functional activities that have a significant strength component.	{1:Strongy disagree}, {2:Disagree}, {3:Agree}, {4:Strongly agree}, {5:I don't know}
If desired, you can make an explanatory statement here:	
New statement: For patients in orthopedic geriatric rehabilitation for whom improving muscle strength is a goal, the effects of muscle strength training can be adequately evaluated by a derived 1RM (e.g.based on 8 or 10 RM) test.	{1:Strongy disagree}, {2:Disagree}, {3:Agree}, {4:Strongly agree}, {5:I don't know}
If desired, you can make an explanatory statement here:	
New statement: For patients in orthopedic geriatric rehabilitation for whom improving muscle strength is a goal, effects of muscle strength training can be adequately evaluated by handheld dynamometry.	{1:Strongy disagree}, {2:Disagree}, {3:Agree}, {4:Strongly agree}, {5:I don't know}

If desired, you can make an explanatory statement here:	
Do you want to make a summary remark regarding TESTING muscle strength in orthopedic geriatric rehabiliation?	
**Part 4: Training muscle strength**	
New statement: The number or repetitions per set is a feasible and valid measure of monitoring muscle strenght training intensity in orthopedic geriatric rehabilitation if you take into account the repetitions in reserve. Repetitions in reserve means that the patient is asked how much repetitions he/she could have additionally performed after a set is completed. Then the load is adjusted to approach the intended intensity (eg. 8 repetitions).	{1:Strongy disagree}, {2:Disagree}, {3:Agree}, {4:Strongly agree}, {5:I don't know}
If desired, you can make an explanatory statement here:	
New statement: There is insufficient scientific evidence for the use of BORG RPE for monitoring strength training intensity in orthopedic geriatric rehabilitation and should not be used.	{1:Strongy disagree}, {2:Disagree}, {3:Agree}, {4:Strongly agree}, {5:I don't know}
If desired, you can make an explanatory statement here:	
Adjusted statement: Even if intensity can not be monitored strength training in orthopedic geriatric rehabilitation is still useful.	{1:Strongy disagree}, {2:Disagree}, {3:Agree}, {4:Strongly agree}, {5:I don't know}
If desired, you can make an explanatory statement here:	
Adjusted statement: The intensity of strentht training in orthopedic geriatric rehabilitation can be adequately monitored during functional activities (eg. By the number of reps in a sit to stand exercise).	{1:Strongy disagree}, {2:Disagree}, {3:Agree}, {4:Strongly agree}, {5:I don't know}
If desired, you can make an explanatory statement here:	
Adjusted statement: Specific and controlled strength training using equipment (eg. a leg press or lat pulley) is NOT necessary to improve muscle strength.	{1:Strongy disagree}, {2:Disagree}, {3:Agree}, {4:Strongly agree}, {5:I don't know}

If desired, you can make an explanatory statement here:	
Adjusted statement: The effect of functional strength training may be further enhanced by combining it with training on specialized equipment.	{1:Strongy disagree}, {2:Disagree}, {3:Agree}, {4:Strongly agree}, {5:I don't know}
If desired, you can make an explanatory statement here:	
Adjusted statement: An intensity of 70-79% of 1RM with a target number of repetitions per set of 7-9 is adequate to improve maximal muscle strength in orthopedic geriatric rehabilitation.	{1:Strongy disagree}, {2:Disagree}, {3:Agree}, {4:Strongly agree}, {5:I don't know}
If desired, you can make an explanatory statement here:	
New statement: An intensity of 40-60% of 1RM with at least 15 repetitions per set is adequate to improve local muscular endurance in orthopedic geriatric rehabilitation.	{1:Strongy disagree}, {2:Disagree}, {3:Agree}, {4:Strongly agree}, {5:I don't know}
If desired, you can make an explanatory statement here:	
Adjusted statement: In order to achieve maximal muscle strength improvement, the training program should continue after orthopedic geriatric rehabiliation, until a total training duration of at least 50 weeks.	{1:Strongy disagree}, {2:Disagree}, {3:Agree}, {4:Strongly agree}, {5:I don't know}
If desired, you can make an explanatory statement here:	
New statement: The strength training guidelines can be tailored to a patients' needs by varying in the interplay between frequency, intensity and session duration, for example by providing more frequent, shorter sessions, or by providing longer sessions of lower intensity.	{1:Strongy disagree}, {2:Disagree}, {3:Agree}, {4:Strongly agree}, {5:I don't know}
If desired, you can make an explanatory statement here:	
New statement: In orthopedic geriatric rehabiliation in most patients a gradual buildup of strength training intensity is necessary to ensure that the patients' technique is correct before intensity is increased to a recommended level.	{1:Strongy disagree}, {2:Disagree}, {3:Agree}, {4:Strongly agree}, {5:I don't know}
If desired, you can make an explanatory statement here:	

Do you want to make a summary remark regarding TRAINING muscle stregnth in orthopedic geriatric rehabiliation?	
Are there any final remarks you wish to make ?	

Round 3

Question	Values
**Part 1: Testing endurance capacity**	
Adjusted statement: For orthopedic geriatric patients that are able to exercise on a (recumbant)cycle ergometer AND are able to talk during low intensity exercise*, the Talk Test is an adequate tool to determine target exercise intensity (in Watts or HR) for endurance capacity training.<br><br>* i.e. not limited by ventilatory or cognitive functioning	{1:Strongy disagree}, {2:Disagree}, {3:Agree}, {4:Strongly agree}, {5:I don't know}
If desired, you can make an explanatory statement here:	
Do you want to make a summary remark regarding TESTING endurance capacity orthopedic geriatric rehabiliation?	
**Part 2: TRAINING endurance capacity**	
Adjusted statement: For orthopedic geriatric patients that are able to exercise on a (recumbant)cycle ergometer AND are able to talk during low intensity exercise*, the exercise intensity for endurance capacity training can be adequately monitored by means of training at a level just below the ventilatory threshold, which means that the patient can just speak in whole sentences.<br><br>* i.e. not limited by ventilatory or cognitive functioning	{1:Strongy disagree}, {2:Disagree}, {3:Agree}, {4:Strongly agree}, {5:I don't know}
If desired, you can make an explanatory statement here:	

Adjusted statement: Endurance capacity training should be continued* after orthopedic geriatric rehabiliation to further increase the effects or to help limit further age-related deterioration.<br><br>*can include daily aerobic physical activities or exercise in different exercise settings e.g. local gym, home based exercise.	{1:Strongy disagree}, {2:Disagree}, {3:Agree}, {4:Strongly agree}, {5:I don't know}
If desired, you can make an explanatory statement here:	
Do you want to make a summary remark regarding TRAINING endurance capacity orthopedic geriatric rehabiliation?	
**Part 3: Testing muscle strength**	
Adjusted statement: For patients in orthopedic geriatric rehabilitation for whom improving muscle strength is indicated as a goal AND resistance exercise is possible, handheld dynamometry is a valuable tool to quantify possible effects of resistance training on local muscle strength, as an addition to more functional evaluation.	{1:Strongy disagree}, {2:Disagree}, {3:Agree}, {4:Strongly agree}, {5:I don't know}
If desired, you can make an explanatory statement here:	
Do you want to make a summary remark regarding TESTING muscle strength in orthopedic geriatric rehabiliation?	
**Part 4: Training muscle strength**	

Adjusted statement: The maximum number of repetitions attained in a set (and compared to the target number of repetitions) is a feasible and valid measure of monitoring muscle strength training intensity in orthopedic geriatric rehabilitation.	{1:Strongy disagree}, {2:Disagree}, {3:Agree}, {4:Strongly agree}, {5:I don't know}
If desired, you can make an explanatory statement here:	
Adjusted statement: The modified BORG RPE scale may be applicable to monitor strength training intensity in orthopedic geriatric patients, without cognitive impairments.	{1:Strongy disagree}, {2:Disagree}, {3:Agree}, {4:Strongly agree}, {5:I don't know}
If desired, you can make an explanatory statement here:	
Adjusted statement: In patients that are not hindered by pain, an intensity associated with 70-79% of 1RM with a target number of repetitions per set of 7-9 is adequate to improve maximal muscle strength in orthopedic geriatric rehabilitation.	{1:Strongy disagree}, {2:Disagree}, {3:Agree}, {4:Strongly agree}, {5:I don't know}
If desired, you can make an explanatory statement here:	
Adjusted statement: Muscle strength improvement can be expected after 6-9 weeks of resistance training and should be continued* after orthopedic geriatric rehabiliation to further increase the effects or to help limit further age-related deterioration.<br><br>* can include daily strength-based physical activities or exercise in different settings e.g. local gym, home-based exercises.	{1:Strongy disagree}, {2:Disagree}, {3:Agree}, {4:Strongly agree}, {5:I don't know}
If desired, you can make an explanatory statement here:	
Do you want to make a summary remark regarding TRAINING muscle stregnth in orthopedic geriatric rehabiliation?	
Are there any final remarks you wish to make ?	
